# Enchondroma in the Bitch1Read before the Montreal Veterinary Medical Association, December 13, 1894.

**Published:** 1895-04

**Authors:** C. A. Boutelle

**Affiliations:** The Pathological Laboratory of the McGill University, Montreal


### ENCHONDROMA IN THE BITCH.[1]

BY C. A. BOUTELLE.

(*From the Pathological Laboratory of the McGill University, Montreal.*)

The study of tumors occurring in the lower animals is one that has been sadly neglected, although it is one of very high interest. So high authorities as Friedberger and Frohner, in their special pathology of higher animals, have not a word to say upon the subject of tumors, and Dieckerhoff only mentions the malignant forms. Williams, in his *Surgery*, refers very briefly to this subject.

Although the references are so brief, it is fully recognized that the dog is frequently afflicted with new growths. Of these perhaps the commonest are in connection with the genital system of the bitch, and of the various regions of this system the mammary glands stand pre-eminent in the frequency with which they are affected. This tendency toward tumor-formation in the dog is peculiarly interesting when we consider that of all animals man is the one most liable to these new growths; also, that of all the lower animals the house-dog is the one most nearly allied to man in its environment, and I might almost say in habits of life.

I will not venture to state whether this exceptional frequency on the part of man and dog is due to the fact that they are both exposed to some common external cause, or whether it is because both in man and dog there is under present conditions a survival of the unfittest; that is to say, that in both man and dog there is a liability for weaklings and those with imperfect bodies (bodies liable to abnormal development) to be preserved alive. We would only suggest there would seem to be some relationship between the habits of existence of man and dog and the relative frequency of tumor development.

[1] Read before the Montreal Veterinary Medical Association, December 13, 1894.

Of tumors in the mammary gland in the bitch the most common are those of the nature of adenomata, or tumors which are of the structure of overgrown gland tissue.

The case I here bring forward is one of a tumor that is, though not rare, not so common, namely, of an enchondroma, or cartilage tumor of this organ. The case is of special interest, inasmuch as the history obtainable was very full, and the cause of events could easily be followed.

The subject was an aged cocker spaniel bitch, which had a litter of puppies in February, 1892. The pups all died from exposure to cold shortly after birth, causing the animal to suffer severely from retention of milk and mammitis.

The owner of the animal endeavored to remove all the milk regularly each day, but in this he was not entirely successful; for some weeks later he noticed a swelling forming in the gland near the posterior teat on the right side. This was hard and painless, and grew slowly until it attained the size of a small orange. In February, 1893, this was removed by Mr. Tracy, a graduate of ('94), with the co-operation of Mr. Hart, a student in the medical faculty of McGill University. The tumor removed at this time was 2½ inches in diameter, and its structure was typical, slightly lobulated and sharply cut off from the surrounding tissues by a dense fibrous capsule.

On section it cut with difficulty, being of cartilage-like density, while toward the centre it was of bony hardness. Sections made by Dr. Adami showed the lobules toward the periphery to be of hyaline cartilage, with some regions presenting a less dense matrix, in which were imbedded some stellate cells. The lobules were obscurely separated by layers of fibrous tissue running down into the cartilage. Further from the surface the matrix became impregnated with calcareous salts, but even at the centre the formation was not quite that of true bone; that is to say, there were large channels in which ran the blood-vessels, but the surrounding solid framework possessed neither proper Haversian canals nor true lamellæ.

This specimen was exhibited by Dr. Adami at a meeting of the Montreal Medico-Chirurgical Society on March 31, 1893 (*Montreal Medical Journal*, vol. xxii., p. 109).

The wound produced by this operation appeared to heal rapidly, and the animal enjoyed the best of health until about the middle of August last. About this time the abdomen was noticed to be enlarged, the bitch having the appearance of pregnancy, except that the mammæ remained small. The enlargement grew rapidly until the first week in October, when the animal was brought to the hospital of the Veterinary College with the view of possible treatment.

Dr. McEachran diagnosed the presence of a tumor, but discountenanced removal on the ground that the great size rendered the survival of the animal after the operation doubtful; while, even if successfully removed, the probability was that similar growths existed in other parts of the system. He recommended that to prevent further suffering the animal be killed at an early date. The owner could not but agree to the mercifulness of this proposal, so on October 18th the bitch was taken to the pathological laboratory of McGill University, and there killed with chloroform.

*Autopsy*. The autopsy was performed immediately after death. On opening up the abdomen a large oval, pale-colored, hard mass was exposed, whose long diameter lay transversely or slightly obliquely across the abdomen. This was apparently enclosed in layers of the peritoneum, and was attached to the omentum and stomach by delicate layers of fibrous tissue containing bloodvessels.

The spleen was firmly attached to the left and undersurface of the mass, but on the anterior surface there could be seen a small portion of hepatic tissue completely detached from the main mass of the liver, about 2½ by 1 inch. The tumor on removal was found to weigh between ten and eleven pounds. The animal when in health had weighed under thirty pounds. On section the outer portion proved in general to be denser than the more central area. The outer portion cut like cartilage, with here and there denser calcareous masses within it. The more central portion was softer and more of a semi-gelatinous nature, and from this there poured a thick, glassy, transparent fluid slightly tinged with blood.

On further examination smaller secondary growths were discovered in the lungs, pancreas, the axillary and mesenteric glands. In the left kidney a small whitish mass was discovered about one-eighth of an inch across, thought to be a secondary growth, but on microscopical examination it was found to be mainly fatty. The heart was normal. At the site of the previous operation a small sinus was discovered, at the base of which was a little growth of glandular rather than of cartilaginous appearance. Portions of the various affected regions were removed and placed in hardening solution for eventual microscopical examination.

*Microscopic examination.* This examination showed, as the naked-eye appearances indicated, the main tumor to be a rapidly growing enchondroma, which had in parts undergone calcareous infiltration, although over a large area the cartilaginous matrix had either been replaced by more mucoid gelatinous material, or through the rapidity of growth proper chondrin had not been developed. In one of the peripheral regions there was present also a considerable number of fat cells. These modifications or accompaniments of other forms of connective-tissue growth are frequent in rapidly growing enchondromata. We have here to deal with, in fact, a lipo-myxo-osteoid enchondroma.

In passing it may here be mentioned that the cells in certain regions were so abundant and closely packed as to give also the appearance of a sarcoma. In other regions there was a fair amount of fibrous tissue; added to this also was an occasional hemorrhage into the substance of the tumor, but these occurrences were apparently rare.

In the affected glands and in the lungs typical small cartilaginous growths were discovered having a tendency to osteoid formation. In the lungs these were numerous, varying from one-sixteenth to one-quarter of an inch in diameter.

The mass at the termination of the sinus in the mammary gland presented a totally different appearance. Here we had to deal with a growth which human pathologists would term either a malignant adenoma or carcinoma.

There was an increase in the fibrous stroma of the region, and in the spaces of the stroma were dilated and very abnormal gland follicles. In the place of a regular and well-developed layer of epithelial cells these follicles were formed of overgrown irregular cells in several layers.

They contained also an inspissated, semi-solid material of a colloid nature, and, what is more, there was to be observed in several of them a papillary ingrowth. Such a condition in the human breast is described as "carcinoma phyllodes, though some authorities like Orth are a little inclined to look upon this as not quite carcinoma, because of the associated cystic dilatation of the follicles. Nevertheless, in this case, there is such definite irregularity in the growth of the epithelium that we cannot look upon this as being carcinomatous.

In the dog it is to be noticed that this condition would not seem to be so highly malignant as it is in man, for in the dog there is not such a liability for secondary growths to develop in other parts of the body following upon mammary tumors of this nature.

There are several features in this case which render it deserving of being placed on record.

First and foremost, we have the development of a cartilaginous tumor in a region which normally contains no trace of this tissue.

The mammary gland is, however, the occasional seat of enchondromata. In man enchondroma of the mammary gland is peculiarly rare. In the bitch it does not seem to be so rare; in fact, this is the second example that has come into the pathological collection at McGill within a year.[1]

Why enchondromata should develop in this region it is most difficult to explain according to any of the present theories of tumor-growth.

Possibly there is some relationship between this and other so-called heteroplastic growths in the mammary gland and their frequent occurrence in the other glandular portions of the genital system; that is to say, it would almost seem as though within the genital apparatus there is a capability or a potentiality for the development of any or every kind of connective-tissue formation. It may be noticed that the testis is another not infrequent seat for the development of enchondromata.

Secondly, this case is remarkable in that here we have such a rich development of metastatic or secondary new growths. This is, to say the least, unusual in the case of enchondromata. These tumors are usually benign, and grow at the seat of origin only. As with other connective-tissue tumors, so with enchondromata, the lung is the most frequent seat of the metastatic tumors, and in this case it is noticeable that the lung was thickly scattered over with small growths of cartilage.

Virchow, in his classical work upon *Tumors*, figures a lung the seat of secondary cartilaginous growths closely resembling the lung in this case. His specimen was also from the bitch.[2]

The reason for this predilection is easily explained. It is

[1] Since writing this article I have observed a third case.

[2] Recently in the case examined by me, since this article was read before the Montreal Veterinary Medical Association, I have come across another example of these pulmonary metastatic growths of cartilage. It would seem probable, therefore, that in the dog there is far greater liability for these metastatic growths to occur than there is in man.

through the bloodvessels that cells are carried away from the primary growth. They are borne by the veins to the right side of the heart and are arrested in the lung capillaries.

Thirdly, some not so arrested may pass through to the left side of the heart, and may be carried to various portions of the system.

It would seem in this case as though the huge secondary growth, which developed in the abdomen, had commenced in the lower portion of one of the lobes of the liver. By no other means do I see how we are to explain the presence upon the surface of the growth of a detached portion of liver tissue. It would appear that in its growth the tumor mass had so pulled upon the lobe in which it was developing, that it eventually became separated and dragged with it the lower portion of the lobe, at the same time gaining secondary vascular supply through the inflammatory adhesions developing around it.

Lastly, it is to be noticed that clearly this tumor developed after disturbed function and injury to the mammary gland.

According to the description given by the owner it was after a condition of mammitis, following upon a retention of milk, that the tumor developed. Such a relationship is far from being uncommon. It is of peculiar interest to notice the sequel in the mammary gland. Evidently the operation wound did not heal completely, but a sinus was left leading down to the mammary tissue, and here again following upon the chronic inflammation there was developed a new growth; but now, instead of this being an enchondroma, the tumor was of the nature of an aberrant gland formation—a malignant adenoma or carcinoma.

---

California will send a delegate to the next meeting of the United States Veterinary Medical Association. This will add another link to the chain of union that tends to encircle through the National Association the entire profession of our country. Among the list of applicants for membership there will be several well known California veterinarians.

Montana, by proclamation, forbids the importation of sheep unless inspected by State Veterinarian and found free from disease, from Oregon, Nevada, California, Washington, Idaho, Colorado, Utah, Okalahoma, and New Mexico.

